# A Novel Pathogenic Variant of the *AVPR2* Gene Leading to Arginine Vasopressin Resistance Since the Neonatal Period

**DOI:** 10.3390/genes16080989

**Published:** 2025-08-21

**Authors:** Agnieszka Szmigielska, Piotr Skrzypczyk, Dorota Czapczak, Marta Dux, Adam Lipka, Beata Pyrżak, Anna Małgorzata Kucharska

**Affiliations:** 1 Department of Pediatrics and Nephrology, Medical University of Warsaw, 02-091 Warsaw, Poland; agnieszka.szmigielska@wum.edu.pl (A.S.); pskrzypczyk@wum.edu.pl (P.S.); 2 Department of Laboratory Diagnostics and Clinical Immunology of Developmental Age, Medical University of Warsaw, 02-091 Warsaw, Poland; dorota.czapczak@wum.edu.pl; 3 Laboratory of Genetics, University Clinical Centre of Medical University of Warsaw, 02-097 Warsaw, Poland; marta.dux@uckwum.pl; 4 Student Scientific Group at the Department of Pediatrics and Nephrology, Medical University of Warsaw, 02-091 Warsaw, Poland; ad_lip@wp.pl; 5 Department of Pediatrics and Endocrinology, Medical University of Warsaw, 02-091 Warsaw, Poland; beata.pyrzak@wum.edu.pl

**Keywords:** diabetes insipidus, arginine vasopressin deficiency, arginine vasopressin resistance, neonate, pathogenic variant

## Abstract

**Background**: Diabetes insipidus (DI) in newborns is an extremely rare condition, with the age of presentation strongly suggesting a genetic background of the disease. The differential diagnosis should include arginine vasopressin deficiency (AVD) and arginine vasopressin resistance (AVR). Some novel diagnostic tools such as copeptin evaluation and genetic tests are vital for early diagnosis. **Case report**: We present the case of a 1-month-old boy with polyuria observed since birth. Laboratory tests showed persistent hypernatremia, elevated plasma and low urine osmolality. An attempt at oral administration of desmopressin had no effect; additionally the copeptin level was increased. A genetic study (NGS of the *AVP*, *AVPR2* and *AQP2* genes) was considered and a new pathogenic variant in the *AVPR2* gene (hemizygous c.157del) was detected. After the genetic test result was obtained, treatment with hydrochlorothiazide was started. The patient is now 3 months old, developing normally, and the weight and height are normal. **Conclusions**: Newborns with DI should be subjected to extensive multidisciplinary diagnostics, including endocrine and renal causes. Copeptin evaluation and prompt genetic diagnosis allows for the early diagnosis and implementation of appropriate treatment.

## 1. Introduction

Diabetes insipidus (DI) in newborns is an extremely rare condition, with the age of presentation strongly suggesting a genetic background of the disease. Two diagnostic directions should be taken into account in such a case: arginine vasopressin deficiency (AVD) or arginine vasopressin resistance (AVR). Inherited AVD affects only 1% of AVD and the majority of cases is acquired condition. The frequency of hereditary AVR is estimated for 1 per 1,000,000 and the cases are associated with pathogenic variants of arginine vasopressin receptor 2 gene, *AVPR2* (90% of cases) or in aquaporin 2 gene, *AQP2* (10% of cases) [1]. A comparison between the frequency of AVD and AVR indicated that in newborns, AVR is more statistically probable than inherited AVD. Nevertheless, the investigation always starts with the evaluation of the symptoms of DI and the response to the desmopressin (DDAVP).

Arginine vasopressin resistance (AVR), previously known as nephrogenic diabetes insipidus (NDI), is defined as a decreased sensitivity of the kidneys to arginine vasopressin (AVP), resulting in an inability to concentrate urine. Congenital cases of AVR are less frequent than the acquired ones, which could be secondary to the intake of certain drugs, such as lithium, as well to electrolyte disturbances (hypokalemia, hypercalcemia) and other renal or systemic diseases such as renal sarcoidosis, sickle cell anemia or polycystic kidney disease [2,3]. Primary congenital AVR is known to be caused by pathogenic variants of *AVPR2* or *AQP2*, genes that encode the type 2 receptor to arginine vasopressin and aquaporin-2 protein, respectively. The loss-of-function mutations in the *AVPR2* gene located on the X chromosome are believed to be responsible for about 90–98% of hereditary AVR cases, with an estimated incidence of approximately 8–8.8 per million male live births [3,4]. The remaining cases result from pathogenic variants of the *AQP2* gene located on chromosome 12, which may be inherited in either an autosomal recessive or dominant manner depending on the variant. The dominant form is less common and typically associated with a milder clinical presentation [3]. Symptoms typically appear during the first year of life, with a median age of 0.6 years according to the study by Lopez-Garcia et al. [5]. These include polyuria and polydipsia, although they may be easily overlooked in infants. Additional symptoms more likely to alert parents are poor feeding, vomiting, fever, growth faltering, constipation and visible signs of dehydration [4,5].

In this case report, we present a 1-month-old boy with symptomatic AVR caused by a previously unreported pathogenic variant of the *AVPR2* gene.

## 2. Case Presentation

### 2.1. Patient Presentation and Clinical Assessment

We present a case of a male child of non-consanguineous, healthy parents, born from the first pregnancy, in the 36th week of gestation +3 days, by spontaneous delivery, and assessed for 10 Apgar points. The pregnancy was complicated by gestational diabetes, well controlled with diet, and hypothyroidism, treated with L-thyroxin oral supplementation. The child’s birth weight was 2830 g, length 48 cm, and head circumference 33 cm. The boy was breastfed, sucked effectively, but constant weight loss was observed from birth. From the first days of life, the parents noticed very abundant diaper wetting. On the 12th day of life, the body weight was 2590 g, and the estimated diuresis was 5–7 mL/kg/hour. Laboratory tests revealed hypernatremia of 158 mmol/L, increased serum osmolality and significantly reduced urine osmolality, without other urinary abnormalities. Capillary blood acid–base balance was normal (pH 7.34, BE (-3.1)). After intravenous rehydration, sodium concentration decreased to 147 mmol/l, and serum osmolality decreased to values slightly above the norm (298 mOsm/kg H_2_O). The boy was discharged home, where he was breastfed on demand for 8 to 12 feedings a day; he was still gaining weight poorly, and he was soaking diapers profusely.

After one week of home observation, follow-up tests again revealed hypernatremia (154 mmol/L), hyperchloremia (120 mol/L) and a normal potassium concentration (4.3 mmol/L). Capillary blood acid–base balance was again normal (pH-7.363, BE (-3.5)), plasma osmolality was substantially increased (311 mOsm/kg H_2_O) and urinalysis revealed the specific gravity of 1002. The boy was admitted to the hospital to the pediatric endocrinology department. The general condition on admission was good; his blood pressure was 88/48 mm Hg. He presented with constant polyuria of 6–8 mL/kg/hour (average water balance: intake +650 mL; output 510 mL). Physical examination showed slight signs of dehydration (dry mucous membranes) and umbilical hernia; otherwise, there were no significant deviations. Laboratory tests showed the following: normal complete blood count, normal glycemia, and liver and kidney function parameters, high serum osmolality (323 mOsm/kg H_2_O) with significantly reduced urinary osmolality (73 mOsm/kg H_2_O) (Table 1), and very high plasma renin (1001 mU/L) and aldosterone (206 ng/dL) concentrations. For the imaging tests, transfontanel and abdominal ultrasound were normal. The magnetic resonance imaging of the hypothalamo-pituitary region described a poorly separated posterior pituitary lobe. Pending the result of copeptin and the genetic test, suspecting AVD and hoping to reduce polyuria, the boy was started on oral desmopressin (lyophilisate) in increasing doses: in the following days, 3.125 µg (1/8 of a 25 µg tablet, Noqturina, Ferring GmbH, Kiel, Germany), then 9.375 µg (3/8 of a 25 µg tablet Noqturina) and 15 µg (1/4 of a 60 µg tablet, Minirin melt, Ferring GmbH, Kiel, Germany) (freeze-dried freshly divided with a scalpel). The administration of desmopressin did not alter serum and urine osmolality (Table 1), and increased diuresis persisted. At the first test, the boy had an extended pause in feedings to 3 h, and during this time, the osmolality (values) of serum and sodium level (154 mmol/L) increased. After 10 days from the blood sampling, the result of copeptin was received: 271 pmol/L. The sample was collected at an osmolality of 310 mOsm/kg H_2_O (normal value: <28.2 pmol/L with an osmolality of 296–300 mOsm/kg H_2_O, according to the adult reference producer’s values) (Table 1). The increased copeptin concentration and no effect after the administration of desmopressin indicated renal insipidus. The genetic tests detected a potentially pathogenic variant of the *AVPR2* gene.

Afterward, the boy remained under nephrological care. He has been receiving hydrochlorothiazide in increasing doses since the age of 2 months (1 mg/kg of body weight/day). The boy is fed exclusively breast milk and supplemented with water, and he gains weight well. The fluid supply is about 200 mL/kg/day; polyuria of 5–7 mL/kg/hour still persists. In follow-up examinations, sodium and potassium concentrations were normal (141 mmol/L and 5.0 mmol/L, respectively), as was serum osmolality, and urine specific gravity remained low: 1003, similar to urine osmolality.

### 2.2. Genetic Diagnosis

#### 2.2.1. Methods

**Figure 1:**
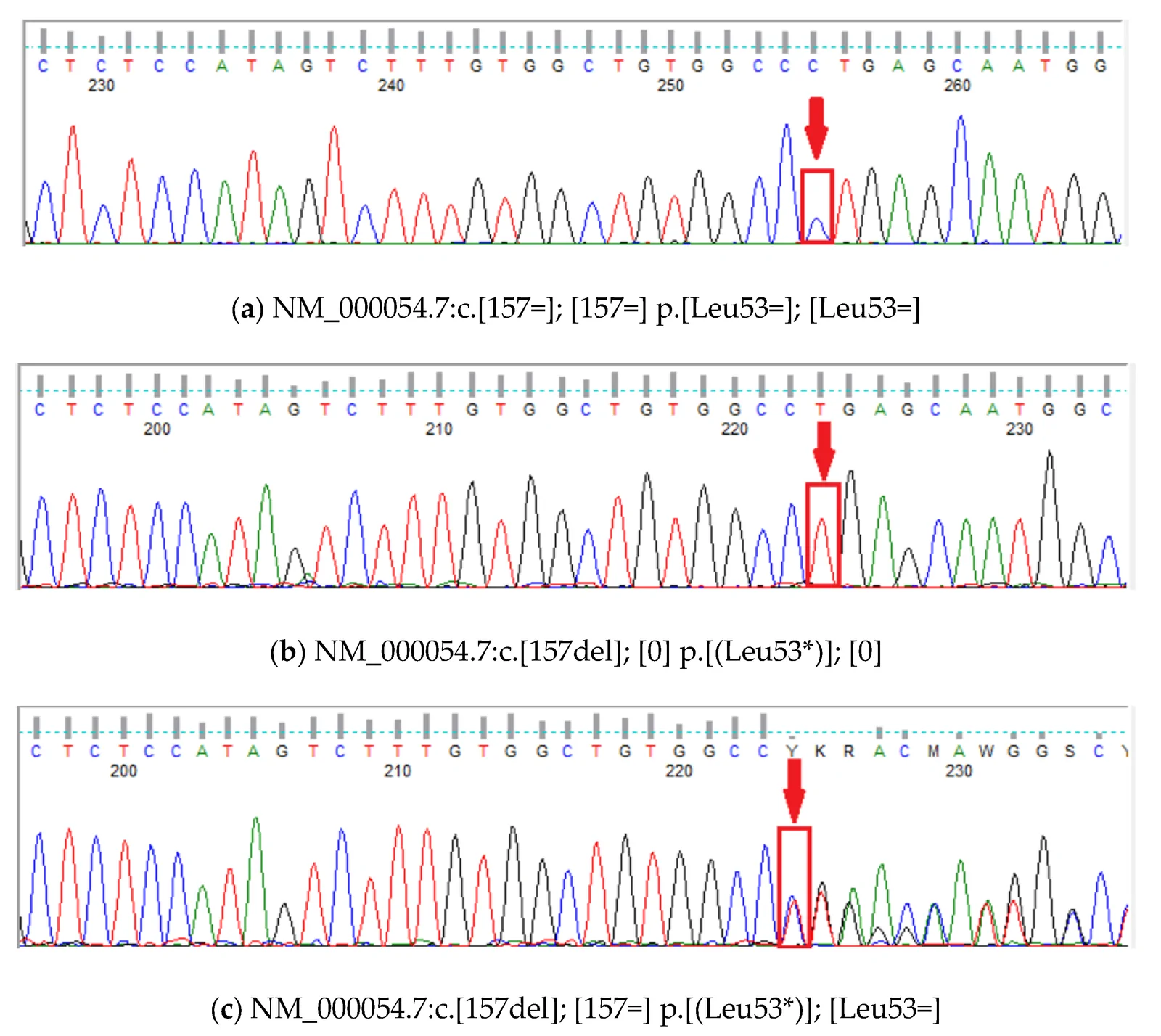
Result of arginine vasopressin receptor 2 *(AVPR2*) gene sequencing. The sequence chromatogram of *AVPR2* in (**a**) the wild type (WT), (**b**) the patient and (**c**) the patient’s mother. In the figures, the deletion site is indicated by the red arrows.

The genomic DNA of the patient and his mother was extracted from whole blood using NucleoSpin Dx Blood (Macherey-Nagel, Düren, Germany). The purity of DNA was checked using a spectrophotometric method on Nanodrop (Thermo Fisher Scientific, Waltham, MA, USA). The DNA was quantified using a fluorometric-based method on Quantus with QuantiFluor ONE dsDNA System (Promega, Madison, WI, USA). The integrity of genomic DNA was determined with a Genomic DNA Reagents Analysis Kit on the TapeStation System (Agilent, Santa Clara, CA, USA). A library of the proband DNA was prepared with AmpliSeq for Illumina On-Demand Panel Custom (Illumina, San Diego, CA, USA) and the testing was performed with the Next-Generation Sequencing method on the MiSeq System (Illumina, San Diego, CA, USA). The detected variant was verified with Sanger sequencing (Figure 1) and analyzed with Mutation Surveyor software [M1]- Version 5.1.0. (Softgenetics, State College, PA, USA).

#### 2.2.2. Genetic Analysis

The analysis revealed a hemizygous variant in the *AVPR2* gene (NM_000054.7:c.157del (p.Leu53Ter)) in the boy and a heterozygous variant in the patient’s mother. The detected variant is not registered in the dbSNP, ClinVar, HGMD-Public or other available databases containing information about mutations in the human genome. There are no information about population variant frequency. The variant is indicated as disease-causing by available algorithms, including in silico pathogenicity predictions (e.g., MutationTaster).

The OMIM database indicates that mutations in the *AVPR2* gene (OMIM *300538) are connected to X-linked nephrogenic diabetes insipidus, NDI1 (OMIM #304800). Loss of function of the *AVPR2* gene is known mechanism of the disease. The detected variant ID is located in 2 of 3 exons coding the gene in the transmembrane domain. The expected effect of the change is leucine at position 53 to a STOP codon and shortening of the protein. It is predicted that the transcript with a premature STOP codon will be eliminated by NMD (nonsense-mediated mRNA decay). Causal variants are most often located in transmembrane domains. Another truncating variant in transmembrane domains has been described as pathogenic in the ClinVar database (RCV004797266.1; NM_000054.7:c.135_136del).

The clinical interpretation of the variant using a classification of the American College of Medical Genetics and Genomics guidelines is likely pathogenic (PVS1, PM2, PP3).

**Figure 2:**
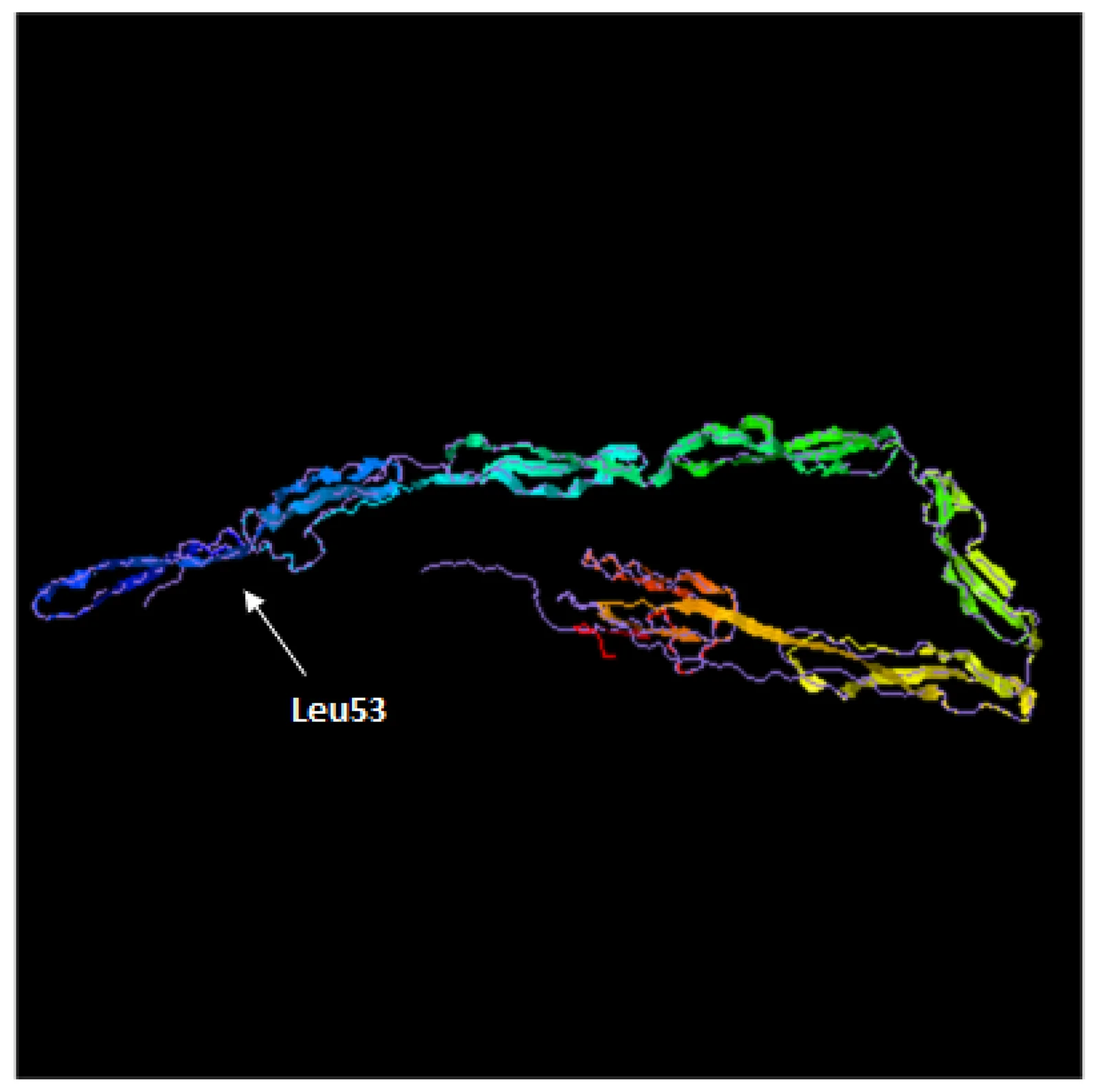
Predicted structural model of arginine vasopressin receptor 2 (AVPR2) protein. The simulation performed using web tool I-TASSER Protein Structure & Functional Predictions (https://doi.org/10.1038/s41587-025-02654-4).

The gene *AVPR2* encodes the vasopressin receptor type 2, also known as the V2 receptor, which belongs to the seven-transmembrane-domain G protein-coupled receptor (GPCR) superfamily, and couples to Gs, thus stimulating adenylate cyclase. The *AVPR2* gene (located on Xq28) encodes a protein of 371 amino acids, characterized by six transmembrane domains connected by five loops, and intracellular N- and C- termini (Figure 2). When the function of this gene is lost, the disease nephrogenic diabetes insipidus (NDI) results, whereas gain-of-function mutations cause nephrogenic syndrome of inappropriate antidiuresis (NSIAD; https://doi.org/10.1016/bs.vh.2019.08.012). The V2 receptor is expressed in the kidney tubule, predominantly in the distal convoluted tubule and collecting ducts, where its primary property is to respond to the pituitary hormone arginine vasopressin (AVP) by stimulating mechanisms that concentrate the urine and maintain water homeostasis in the organism. X-linked NDI associated with variants in the *AVPR2* gene mostly affects males, with variable penetrance in heterozygous women, depending on the presence of inactivated X bias.

## 3. Discussion

We present the case of a boy who had the symptoms of diabetes insipidus (polyuria, hypernatremia, high plasma osmolality with low urine osmolality) since birth. Molecular studies revealed a new, previously undescribed pathogenic variant of the AVPR2 gene, producing an inactive protein responsible for the child’s phenotype. The efficient cooperation between an endocrinologists, nephrologists and geneticists allowed for making an early diagnosis and initiating treatment.

Until 2022, the traditional terms for central diabetes insipidus caused by vasopressin deficiency and nephrogenic diabetes insipidus, resulting from a lack of sensitivity of the renal tubules to vasopressin, were used. In 2022, the Working Group for Renaming Diabetes Insipidus changed the names of these diseases to arginine vasopressin deficiency (AVD) and arginine vasopressin resistance (AVR) to name these two entities according to their true pathophysiologic mechanism [6].

Children with AVR usually come from a normal pregnancy, and polyuria and consequently polyhydramnios are not typical symptoms of the disease [7]. According to large registries, the first symptoms usually become evident later than in our patient, around 3–4 months of age [5,8]. Although infants are generally more prone to dehydration, in the case of children with AVR, life-threatening dehydration usually occurs only after switching to solid foods, due to their greater osmotic load compared to mother’s milk or milk-replacing formulas. It is worth emphasizing that our patient presented severe polyuria with typical biochemical disorders from the first days of life.

Our patient presented typical biochemical disorders: high plasma osmolality caused by hypernatremia, simultaneous low (always lower than plasma) urine osmolality and polyuria. No other biochemical disorders were found in the patient that could have suggested other tubulopathies (e.g., renal tubular acidosis or Bartter syndrome); potassium, calcium, and magnesium levels were normal, and the patient had normal calcium-phosphate metabolism [7,9].

According to the 2025 international expert consensus statement, the detection of inappropriately diluted urine (i.e., urinary osmolality <200 mOsm/kg H_2_O), combined with high–normal or elevated serum sodium, is pathognomonic for the diagnosis of diabetes insipidus (nephrogenic or central) and warrants early genetic testing [7]. Possible central, renal and other causes of polyuria in neonates and infants are presented in Table 2.

Among patients with genetically determined AVR, 90% of patients have a pathogenic variant in the *AVPR2* gene, which is located on the long arm of the X chromosome (Xq28) (OMIM #304800), hence symptoms occur in boys. In girls, normally one of the two X chromosomes in each cell is randomly inactivated early in development. This inactivation occurs with either the maternally or paternally inherited X chromosome being inactivated with a roughly equal probability in each cell. Therefore, some women with a pathogenic variant of the *AVPR2* gene may also develop the disease as a consequence of X inactivation of the chromosome carrying the normal variant. The remaining 10% of AVR patients have pathogenic variants of the *AQP2* gene encoding aquaporin 2; in these patients (of both sexes), there may be autosomal recessive (OMIM #222000) or dominant (OMIM #125800) inheritance, and symptoms may be milder than in the X-linked form [7,10]. Of note, many acquired conditions may cause the renal tubule resistance to AVP. These conditions are primarily seen in older children and adults (Table 3).

Following the 2025 consensus, we performed a genetic test on the patient as soon as possible using the next-generation sequencing method for three genes: *AVP*, *AVPR2* and *AQP2* [7]. We found a new, previously undescribed pathogenic variant of the *AVPR2* gene in the patient under examination. The variant (c.157del) causes the shortening and complete inactivation of the protein. The complete inactivity of the receptor in the absence of a second correct copy of the gene explains the severe phenotype observed in the boy, unlike in most patients with AVR, from the first days of life [10].

The recommendations emphasize that other tests differentiating polyuria should be performed in the case of a long wait for a genetic test, the impossibility of performing it or a negative result of genetic studies [7]. Copeptin is secreted together with AVP in equimolar amounts. In contrast to AVP, it is characterized by stability. The concentration of copeptin has already been assessed in various clinical settings, including children with nocturnal enuresis [17], primary hypertension [18] and chronic kidney disease [19]. In adults, the cut-off point is assumed to be 21.4 pmol/L, without such standards for children. In a patient with polyuria, the concentration of copeptin >21.4 pmol/L indicates insensitivity of the tubule to vasopressin [20]. It is worth emphasizing that the concentration of copeptin in our patient was about ten times higher than the cut-off point, which is further evidence of the peripheral background of polyuria.

The tests assessing the hypothalamic–renal system are the water deprivation and DDAVP tests. DDAVP is a synthetic analog of AVP that does not affect the V1 receptor. The drug has been widely used in the treatment of AVR and primary nocturnal enuresis for many years. The DDAVP test should be performed carefully, ensuring full control over the child’s condition. In the case of a normal tubular response to DDAVP, there may be sudden water retention and a rapid decrease in serum sodium, which may have adverse consequences for the central nervous system [7]. In our patient, while waiting for the genetic test result, a DDAVP test was performed. A commercially available oral preparation was administered in gradually increasing doses in controlled, hospital conditions. We did not observe any side effects, the child’s condition did not change, and there was no effect on biochemical disorders, which is another confirmation of the diagnosis. We did not perform a water deprivation test for obvious reasons, such as patient safety.

Treating a child with AVR is difficult and requires very good cooperation with the family. The period of infancy and early childhood is particularly dangerous because the patient does not articulate his or her needs regarding fluids. In addition, due to the body’s higher physiological content of water (70% vs. 60%), dehydration occurs more easily at this age. In the youngest children, it is recommended to flush with water, and if intravenous hydration is necessary, unlike in the general population, a 5% glucose solution is recommended to avoid an additional sodium load [21]. For infants, breast milk or a traditional formula milk substitute is recommended. According to the literature data, about one-fourth of children with AVR may require tube feeding or gastrostomy in early childhood [5,8,22]. Our patient is gaining adequate weight on mixed feeding. We will closely monitor his further development.

In patients with persistent hypernatremia and a tendency for dehydration, also in infancy, it is recommended to include drugs that limit diuresis: thiazide/thiazide-like diuretics and cyclooxygenase inhibitors. The mechanism of action of thiazide diuretics is unclear. It is postulated that these drugs cause a small intravascular depletion, which causes increased reabsorption of sodium and water in the initial parts of the tubules, as a result of which less water enters the collecting tubules and less urine is produced. Patients treated with thiazide diuretics are at a risk of developing biochemical complications: fluctuations in sodium concentration to hyponatremia, hypokalemia, hypercalcemia and hyperuricemia. In our patient, we used hydrochlorothiazide as wafers in gradually increased doses starting from 0.5 mg/kg/day. Non-pharmacological treatment together with pharmacological treatment allowed for the maintenance of normonatremia, proper hydration, and nutrition status in our patient despite persistent polyuria.

In our patient, hydrochlorothiazide had a reduced urine output by blocking the sodium–chloride co-transporter in the distal convoluted tubule and allowed for controlling the level of sodium in blood. The loss of potassium caused by thiazide diuretics, may require adding of potassium-sparing diuretics, such as amiloride, to the treatment, but in most cases, a high-potassium diet allow for avoiding hypokalemia. Additionally, reducing salt intake potentiates the efficacy of diuretics. NSAIDs (ibuprofen or indomethacin) can be used in combination with diuretics in NDI, but the tolerance of indomethacin is poor because of the gastric side effects. Patient control includes the monitoring of growth and electrolyte balance. Height and weight are the key parameters at each follow up for children. The decision of treatment with thiazide diuretics or combined treatment with NSAIDs or amiloride depends on growth and laboratory tests (serum electrolyte levels, and serum and urine osmolality).

Large international registries assessing long-term prognosis in patients with AVR have shown that the most commonly used drugs in this group of patients are thiazide/thiazide-like diuretics and potassium-sparing diuretics (approx. 70% of patients), and less frequently NSAIDs (less than 50% of patients). Over time, there is an improvement in height and weight expressed in SDS. Most adults have a normal height, but an increased prevalence of obesity was noted. Urological complications (hydronephrosis, bladder dysfunction with residual urine volume) occur in approximately 40% of patients. Chronic kidney disease in stage 2 or higher was revealed in as many as 32% of children and half of adults [5,8]. Moreover, it could have delayed unpredictable consequences in the function of metabolic and immune balance, i.e., protein modifications and associated immune responses have been implicated in the pathophysiology of other chronic endocrine and metabolic disorders such as type 2 diabetes mellitus, where glycoxidatively modified IgG can trigger autoantibody formation and contribute to disease progression [23].

## 4. Conclusions

We presented a case of an infant with polyuria occurring from the first days of life, and indicated that such a situation requires urgent medical intervention. First, serum sodium concentration, plasma osmolality and urine should be assessed to confirm or exclude diabetes insipidus. At the next stage, distinguishing between arginine vasopressin resistance and deficiency is necessary. In this process, copeptin evaluation and genetic tests play a vital role. In the described boy, we detected a new pathogenic variant of the *AVPR2* gene, resulting in early-onset AVR managed with careful non-pharmacological and pharmacological treatment. The cooperation between specialist clinicians and the genetic laboratory is crucial for the early diagnosis and implementation of proper treatment.

## Figures and Tables

**Table 1:** Electrolyte values, and serum and urine osmolality in the patient during diagnostics.

Age [day]	Circumstance	Sodium [mmol/L]	Potassium [mmol/L]	Serum Osmolality [275–294 mOsm/kgH_2_O]	Urine Osmolality [275–900 mOsm/kgH_2_O]	Urine Gravity [g/mL]
12	In the department of neonatology	158	4.7	315	66	1003
21	After i.v. rehydration	147	5.4	298.1	60	1003
28	After 7 days at home	154	5.0	310	43	1002
29	Admission to the hospital	157	5.3	323	73	1001
31	After i.v. rehydration	145	5.1	298	60	1002
34	Copeptine sampling (271 pmol/L) *	147	5.4	310	73	1001
35	After DDAVP sublingualy (3.125 µg) + 3 h without feeding	154	5.4	320	66	1002
36	After DDAVP sublingualy (9.375 µg)	151	5.1	310	43	1001
37	After DDAVP sublingualy 15 µg	154	5.1	310	66	1001

* Sample was obtained at the age of 34 days, and the result received after 10 days. DDAVP—desmopressin lyophilisate.

**Table 2:** Central, renal and other possible causes of polyuria and polydipsia in infants and neonates.

Central Etiology	Renal Etiology	Other
Arginine vasopressin deficiency (formerly, central diabetes insipidus)	Arginine vasopressin resistance (formerly, nephrogenic diabetes insipidus)	Congenital adrenal hyperplasia with salt wasting
Cerebral salt wasting syndrome	Chronic kidney disease of different etiologies (especially congenital anomalies of the kidney and urinary tract, nephronophthisis, ciliopathies)	Diabetes mellitus
	Bartter syndrome (especially types 1, 2, and 5)	Hypercalcemia (e.g., vitamin D intoxication, idiopathic infantile hypercalcemia)
	Congenital or acquired Fanconi syndrome	Hypokalemia (e.g., familial hyperaldosteronism)
	Distal renal tubular acidosis	
	Apparent mineralocorticoid excess	

**Table 3:** Possible causes of arginine vasopressin resistance (AVR) according to the literature data [7,9,11,12,13,14,15,16].

Congenital	Acquired
Pathogenic variants in the arginine vasopressin receptor 2 *(AVPR2*) gene Pathogenic variants of the aquaporine-2 (*AQP-2*) gene Sickle cell disease Cystinosis Bartter syndrome types 1, 2, and 5 Familial hypomagnesemia with hypercalciuria Nephrocalcinosis (different etiologies) Ciliopathies: autosomal recessive/dominant polycystic kidney disease, nephronophthisis, Bardet–Biedl syndrome)	Chronic kidney disease (different etiologies) Recovery from acute kidney injury Bilateral urinary tract obstruction Renal tissue infiltration (sarcoidosis, amyloidosis, hemochromatosis, Sjögren syndrome, multiple myeloma) Ion disturbances (hypercalcemia, hypokalemia) Medications (lithium, foscarnet, ifosfamide, demeclocycline, cisplatin, carboplatin, aminoglycosides, amphotericin B, methoxyflurane, sevoflurane, vinblastine, cyclophosphamide, colchicine, sulfonylureas, ofloxacin, didanosine, cidofovir)

## Data Availability

The original contributions presented in this study are included in the article. Further inquiries can be directed to the corresponding authors.
